# Retrospective study on the efficacy of monocyte distribution width (MDW) as a screening test for COVID-19

**DOI:** 10.1186/s40001-023-01086-7

**Published:** 2023-03-27

**Authors:** Kentaro Wakamatsu, Zenzo Nagasawa, Kouta Katsuki, Hiroyuki Kumazoe, Masayo Yasuda, Sae Kawamoto, Ayano Kawamura, Tsuyoshi Ueno, Ruriko Kiyotani, Izumi Fukui, Sanae Maki, Nobuhiko Nagata, Masayuki Kawasaki, Hozumi Yamada

**Affiliations:** 1 Department of Respiratory Medicine, National Hospital Organization Omuta National Hospital, 1044-1 Oaza, Tachibana, Omuta City, Fukuoka 837-0911 Japan; 2 Department of Medical Technology and Science, Faculty of Fukuoka Health Care, International University of Health and Welfare, 137-1 Enokizu, Okawa City, Fukuoka 831-8501 Japan; 3 Department of Clinical Laboratory, National Hospital Organization Omuta National Hospital, 1044-1 Oaza, Tachibana, Omuta City, Fukuoka 837-0911 Japan; 4 Department of Respiratory Medicine, Fukuoka Sanno Hospital, 3-6-45 Momochihama, Sawara-ku, Fukuoka City, Fukuoka 814-0001 Japan; 5 Department of Respiratory Medicine, Keitendo Koga Hospital, 1150 Kamioda, Kohoku Town, Kishima Gun, Saga 849-0506 Japan

**Keywords:** Coronavirus disease 2019 (COVID-19), Severe acute respiratory syndrome coronavirus 2 (SARS-CoV-2), Monocyte distribution width (MDW), DxH 900, Monocyte

## Abstract

**Background:**

Pathogenic genetic testing for coronavirus disease 2019 (COVID-19) can detect viruses with high sensitivity; however, there are several challenges. In the prevention, testing, and treatment of COVID-19, more effective, safer, and convenient methods are desired. We evaluated the possibility of monocyte distribution width (MDW) as an infection biomarker in COVID-19 testing.

**Methods:**

The efficacy of MDW as a screening test for COVID-19 was retrospectively assessed in 80 patients in the COVID-19 group and 232 patients in the non-COVID-19 group (141 patients with acute respiratory infection, 19 patients with nonrespiratory infection, one patient with a viral infection, 11 patients who had received treatment for COVID-19, one patient in contact with COVID-19 patients, and 59 patients with noninfectious disease).

**Results:**

The median MDW in 80 patients in the COVID-19 group was 23.3 (17.2–33.6), and the median MDW in 232 patients in the non-COVID-19 group was 19.0 (13.6–30.2) (*P* < 0.001). When the COVID-19 group was identified using the MDW cut-off value of 21.3 from the non-COVID-19 group, the area under the curve (AUC) was 0.844, and the sensitivity and specificity were 81.3% and 78.2%, respectively. Comparison of MDW by severity between the COVID-19 group and patients with acute respiratory infection in the non-COVID-19 group showed that MDW was significantly higher in the COVID-19 group for all mild, moderate I, and moderate II disease.

**Conclusions:**

MDW (cut-off value: 21.3) may be used as a screening test for COVID-19 in fever outpatients.

*Trial registration* This study was conducted after being approved by the ethics committee of National Hospital Organization Omuta National Hospital (Approval No. 3-19). This study can be accessed via https://omuta.hosp.go.jp/files/000179721.pdf.

## Background

Coronavirus disease 2019 (COVID-19) is caused by severe acute respiratory syndrome coronavirus 2 (SARS-CoV-2), and cases of COVID-19 have been reported globally. The World Health Organization (WHO) declared a pandemic in March 2020. In Japan, it is widely known as an unprecedented infection in the twenty-first century. For example, the government issued a declaration of emergency depending on the increase in the number of infected people and the degree of strain on medical institutions.

In the prevention, testing, and treatment of COVID-19, more effective, safer, and convenient methods are desired. Pathogenic genetic testing by polymerase chain reaction (PCR) was performed to confirm the presence or absence of SARS-CoV-2. Pathogenic genetic testing for COVID-19 can detect viruses with high sensitivity; however, there are several challenges. For example, dedicated analytical equipment and skilled human resources are needed, it takes several tens of minutes to several hours to obtain results, and the reagent cost per test is relatively high.

In recent years, remarkable progress has been made in automated hematology analyzers for laboratory testing, and recent hematology analyzers can be used to analyze not only complete blood count (CBC) (including red blood cell (RBC) count, white blood cell (WBC) count, and platelet count parameters) and WBC differentiation, but also detailed information on WBC. WBC analysis was performed using an automated hematology analyzer, UniCel DxH 900 Series Coulter Cellular Analysis System (Beckman Coulter, K.K.; DxH 900), cell volume and internal cell structure were analyzed based on detecting and measuring changes in electrical resistance and scattered laser light of flow cytometry and conductivity of cells. These parameters are also utilized as numerical values. Since WBC analysis by an automated hematology analyzer can be performed in approximately one minute, clinical laboratories use this analysis as a daily routine examination in hematology testing. Monocyte distribution width (MDW) is a unique parameter of DxH 900 showing monocyte volume distribution width. In the US, DxH 900, which measures MDW as an Early Sepsis Indicator, has been approved by the United States (US) Food and Drug Administration (FDA) as a device to assess risk for sepsis. In Europe, DxH 900 has received a Conformité Européenne (CE) mark. MDW has been attracting attention as a new biomarker. There are reports indicating that MDWs are useful for the early diagnosis of sepsis in the emergency department and for differentiating systemic inflammatory response syndrome (SIRS) and sepsis [1–8].

In severe infections such as sepsis, cytokine storms and activation of WBCs are known to occur [9–13], and morphological changes in monocytes [14] and cytokine storms have been reported in COVID-19, which is an infectious disease [15, 16]. Therefore, the usefulness of MDW as an index of COVID-19 has been reported [17–19].

In this study, to explore the possibility of MDW as an infection biomarker in COVID-19 testing, we analyzed MDW in the COVID-19 group by severity, including mild, moderate I, and moderate II [20] severity, and MDW in the non-COVID-19 group as a comparator and evaluated the clinical usefulness of MDW.

## Methods

### Patient enrollment

Among 88 patients diagnosed with COVID-19 by PCR testing among patients who visited National Hospital Organization Omuta National Hospital between April and September 2021, 80 patients whose MDW was measured on hospital admission (severity: mild: 18 patients, moderate I: 46 patients, and moderate II: 16 patients) were included in the study. This study also included 232 people who were suspected to have COVID-19 or were diagnosed with non-COVID-19 based on SARS-CoV-2 PCR screening testing before hospital admission. In the study, a comparative evaluation was performed retrospectively using blood samples collected at the initial visit. Severity was classified as mild, moderate I, and moderate II according to the Medical Practice Guidelines Version 6.2 [20] and severity of non-COVID-19 was classified according to the same criteria. The details of the patient background are shown in Tables 1 and 2.

**Table 1:** Background of the COVID-19 group

	Age	Male/female	WBC (μL)	CRP (mg/dL)	MDW
Total (*N* = 80)	49 (15–87)	45/35	4600 (2300–12,500)	1.65 (0.02–25.48)	23.3 (17.2–33.6)
Mild (*N* = 18)	35 (15–72)	7/11	4600 (2300–7700)	0.65 (0.06–3.62)	23.0 (17.2–29.0)
Moderate I (*N* = 46)	41 (18–87)	26/20	4600 (2400–9500)	1.64 (0.02–10.42)	22.6 (18.1–33.6)
Moderate II (*N* = 16)	64.5 (54–82)	12/4	5000 (2500–12,500)	7.81 (0.38–25.48)	26.8 (22.2–33.0)

Data are expressed as the median (range)

WBC, white blood cell; CRP, C-reactive protein; MDW, monocyte distribution width

**Table 2:** Background of the non-COVID-19 group

	Age	Male/female	WBC (μL)	CRP (mg/dL)	MDW
Total (*N* = 232)	70 (13–96)	109/123	6700 (500–21,100)	0.565 (0.01–31.46)	19.0 (13.6–30.2)
Acute respiratory infection (*N* = 141)	69 (15–92)	67/74	7200 (500–21,100)	1.59 (0.01–31.46)	19.4 (15.0–28.2)
Mild (*N* = 80)	54.5 (15–92)	33/47	6550 (500–21,100)	0.37 (0.01–13.64)	18.9 (15.0–27.2)
Moderate I (*N* = 52)	77.5 (15–90)	30/22	9350 (2100–17,900)	3.96 (0.46–31.46)	20.0 (16.4–28.2)
Moderate II (*N* = 9)	79 (35–91)	4/5	6500 (3700–13,800)	9.21 (0.46–22.55)	21.2 (16.5–25.0)
Nonrespiratory infection (*N* = 19)	64 (13–87)	7/12	8400 (3800–18,100)	5.01 (0.03–23.15)	23.0 (16.6–30.2)
Viral infection (*N* = 1) (generalized herpes zoster)	81	0/1	3100	1.84	27.7
After treatment for COVID-19 (*N* = 11) [median time from onset to testing: 43 days (11–140 days)]	68 (24–75)	7/4	5500 (2600–9000)	0.09 (0.02–0.7)	18.1 (16.6–21.7)
Contacted with COVID-19 patients (*N* = 1)	21	1/0	6100	0.01	16.2
Noninfectious disease (*N* = 59)	72 (17–96)	26/33	5400 (3200–12,800)	0.11 (0.01–6.5)	18.0 (13.6–21.7)

Data are expressed as the median (range)

WBC, white blood cell; CRP, C-reactive protein; MDW, monocyte distribution width

In the diagnosis of COVID-19, nasopharyngeal swabs were collected from patients, and the samples were analyzed by a fully automated genetic analyzer, Smart Gene (Mizuho Medy, Co., Ltd.). In addition, MDW in EDTA 2 K-added whole blood venous samples was measured by a DxH 900 (Beckman Coulter, K.K.

Pneumonia was diagnosed by two radiologists and one pulmonologist based on chest computed tomography (CT) findings at the initial visit to the hospital.

This study was conducted after being approved by the ethics committee of National Hospital Organization Omuta National Hospital (Approval No. 3-19).

### Detection method of SARS-CoV-2 with Smart Gene (nasopharyngeal swab samples)

The Smart Gene reagent consists of test cartridges with all reagents required for purification, amplification, and detection of nucleic acids and dedicated extraction reagent solution. Analysis was performed according to the manufacturer’s instructions. Nasopharyngeal swabs were collected from the patients using the sterilized cotton swabs recommended by the kit (Nipro sponge swab TYPE R, Nipro Corporation), and they were suspended with dedicated extraction reagent solution. Then, four drops (approximately 110 μL) of the suspended sample were dripped into a test cartridge, and analysis was performed using Smart Gene. Nucleic acid purification, reverse transcription reaction, and PCR were performed for up to 45 cycles. The sample was defined as positive when the result exceeded the threshold from 23 cycles of PCR [21–23].

### Evaluated items at the initial hospital visit

At the initial visit, age, sex, presence or absence of pneumonia, WBC, MDW, C-reactive protein (CRP), and oxygen saturation of the peripheral artery (SpO2) were evaluated.

### Evaluation of diagnostic performance for COVID-19 using MDWs

A receiver operating characteristic (ROC) analysis on the usefulness of MDW was performed in the COVID-19 group and the non-COVID-19 group. The non-COVID-19 group consisted of patients suspected of having COVID-19 who were diagnosed with non-COVID-19 based on SARS-CoV-2 PCR screening testing before hospital admission.

### Discrimination performance of MDWs between COVID-19 and acute respiratory infection

MDW values were compared by severity between the COVID-19 group and patients with acute respiratory infection in the non-COVID-19 group.

### Assessment of factors associated with MDW

Since the patients in the COVID-19 group were younger than the patients with acute respiratory infection in the non-COVID-19 group, a multiple regression analysis was performed for factors associated with MDW in these two groups to exclude age.

### Predictors of COVID-19

Multivariate analyses were performed to assess clinical parameters to distinguish between COVID-19 patients and non-COVID-19 acute respiratory infection patients. In addition, an ROC analysis was performed on the remaining parameters as COVID-19 predictors in the multivariate analysis.

### Statistical analysis

Data are expressed as medians and quartiles, and a nonparametric analysis was performed since the data distribution was not normal. The Mann‒Whitney *U* test was used to test two groups, and the Kruskal‒Wallis test was used to test three and four groups. Diagnostic performance for COVID-19 and non-COVID-19 was assessed by an ROC analysis. A multiple regression analysis was performed for factors associated with MDW. In the significance test, *P* < 0.05 was regarded as statistically significant. All statistical analyses were performed using Excel statistics, Bellcurve for Excel (version 3.21, Social Survey Research Information Co., Ltd.

## Results

### Background of the COVID-19 group

**Fig. 1:**
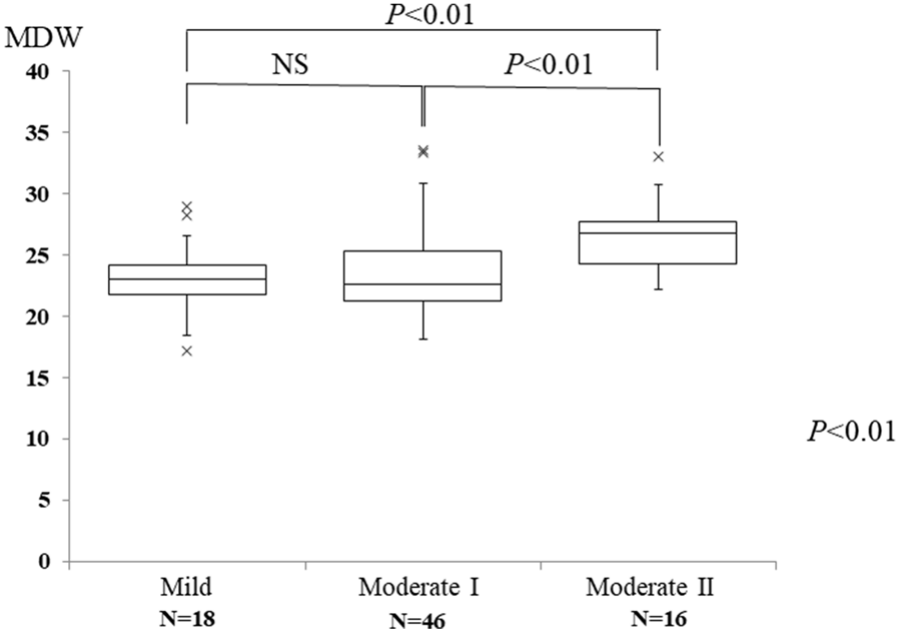
Relationship between MDW and severity in COVID-19 patients. Data are expressed as the median and interquartile range (box). × shows boxplot outliers. MDW, monocyte distribution width

The median age was 49 years, and the disease was more severe with increasing age. CRP and MDW increased with increasing severity (Table 1). MDW was not significantly different between the mild and moderate I patients, but was statistically higher in the moderate II patients than in the mild and moderate I patients (*P* < 0.01) (Fig. 1).

### Background of the non-COVID-19 group

**Fig. 2:**
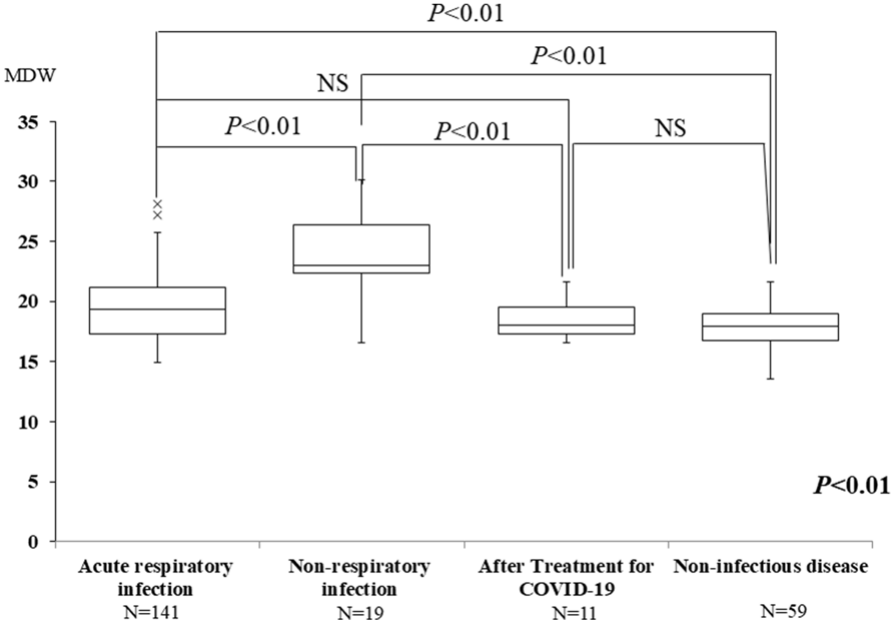
Comparison of MDWs in non-COVID-19 patients. Data are expressed as the median and interquartile range (box). × shows boxplot outliers. MDW, monocyte distribution width

The non-COVID-19 group included 141 patients with acute respiratory infection, 19 patients with nonrespiratory infection, one patient with a viral infection (generalized herpes zoster), 11 patients who had received treatment for COVID-19 [median time from onset to testing: 43 days (11–140 days)], one patient who was in contact with COVID-19 patients, and 59 patients with noninfectious disease (Table 2). The median age was 70 years of age in the non-COVID-19 group, which was higher than that in the COVID-19 group. In general, CRP and MDW showed increased values in infectious disease, and statistically significant differences were observed among the four groups of acute respiratory infections, nonrespiratory infections, the patients who had received treatment for COVID-19, and noninfectious disease (*P* < 0.01). Although there was no significant difference between the patients with acute respiratory infection and the patients who had received treatment for COVID-19, MDW was statistically higher than in the noninfectious disease group. MDW was statistically higher in the nonrespiratory infection group than in the other groups, and the results showed no significant difference between the group of patients who had received treatment for COVID-19 and the nonrespiratory infection group (Fig. 2).

### Evaluation of diagnostic performance for COVID-19 using MDW

**Fig. 3:**
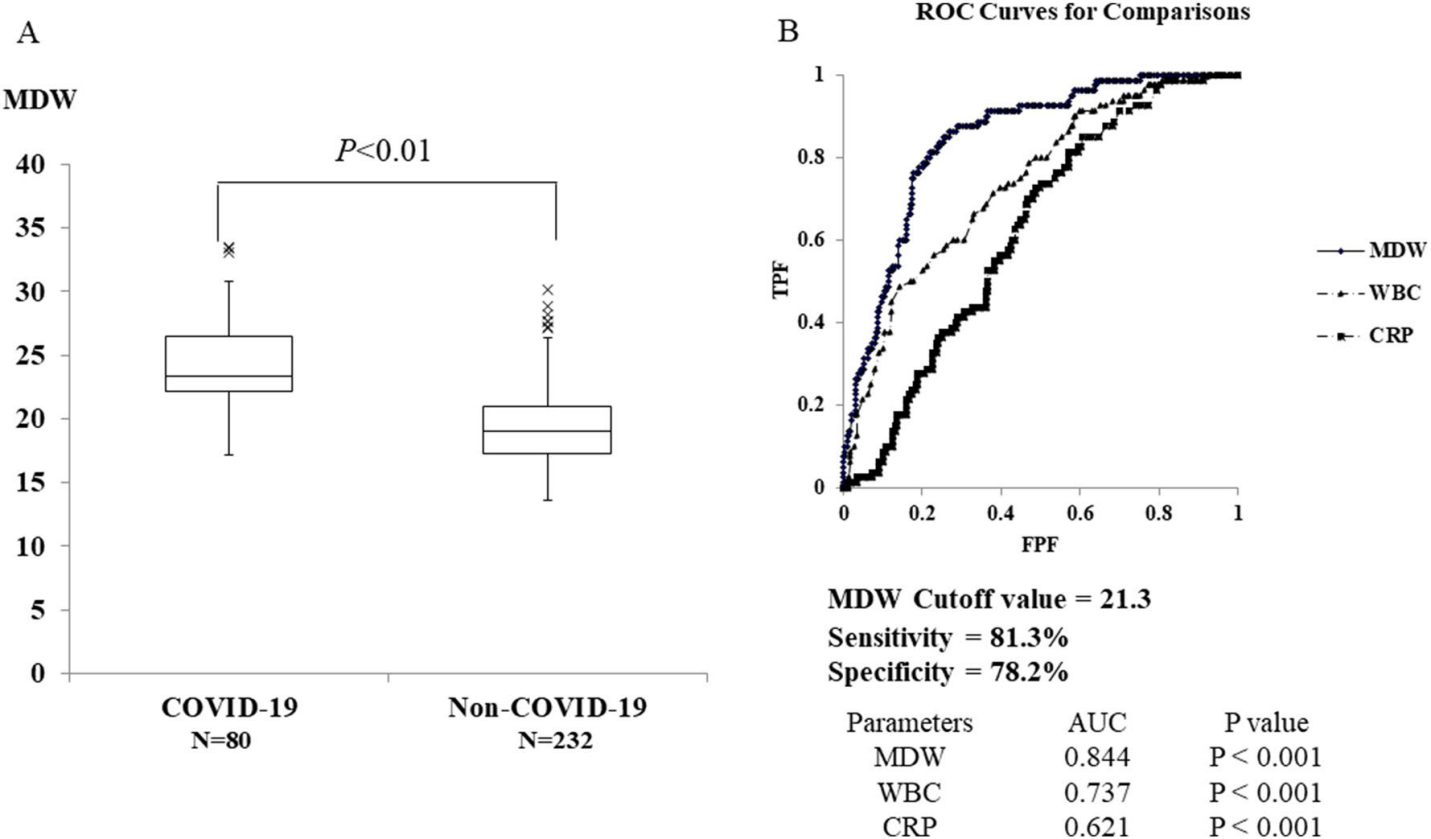
**a**, **b** The results of the ROC analysis

An ROC analysis was performed to evaluate the diagnostic performance of MDW, CRP, and WBC count in the COVID-19 group and the non-COVID-19 group. The results revealed that MDW (area under the curve (AUC): 0.844) had higher diagnostic performance than WBC (AUC: 0.737) and CRP (AUC: 0.621). At the MDW cut-off value of 21.3, the sensitivity and specificity were 81.3% and 78.2%, respectively (Fig. 3).

### Background of acute respiratory infection in the non-COVID-19 group

**Fig. 4:**
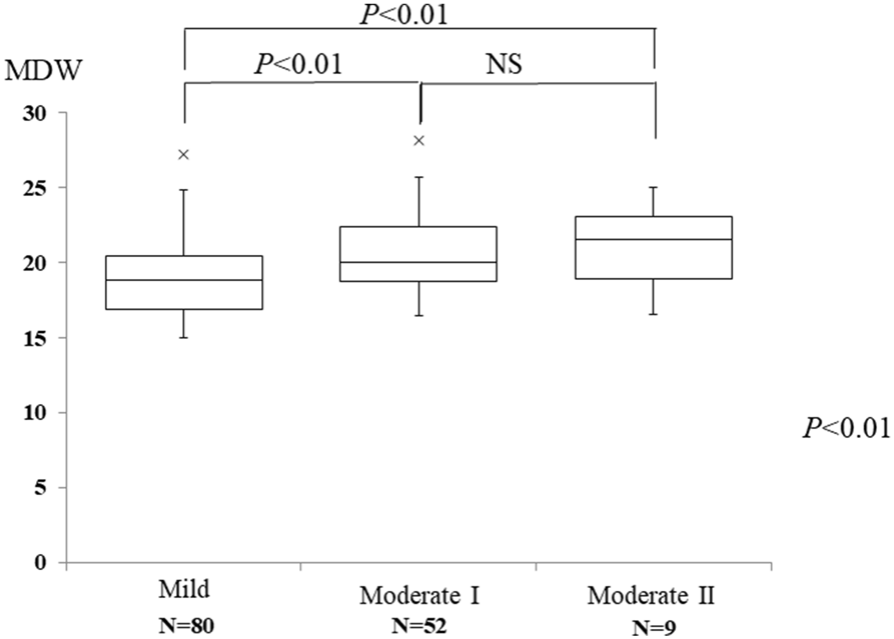
Relationship between MDW and severity in non-COVID-19 patients with acute respiratory infection. Data are expressed as the median and interquartile range (box). × shows boxplot outliers. MDW, monocyte distribution width

The non-COVID-19 group included 80 patients with a mild infection, 52 patients with a moderate I infection, and nine patients with a moderate II infection. The median age was 69 years in the non-COVID-19 group, which was higher than that in the COVID-19 group (Table 2). CRP and MDW increased with increasing severity. Since the number of moderate II patients was small, MDW was not significantly different between the moderate I and moderate II patients (*P* < 0.01). However, MDW was significantly higher in the moderate I and moderate II patients than in the mild patients (Fig. 4).

### Discrimination performance of MDWs between COVID-19 and acute respiratory infection

**Fig. 5:**
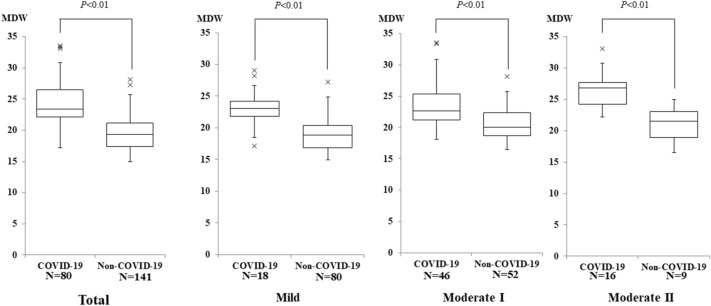
Comparison of MDW by severity between COVID-19 patients and non-COVID-19 patients with acute respiratory infection. Data are expressed as the median and interquartile range (box). × shows boxplot outliers. MDW, monocyte distribution width

Since the MDW value increased with increasing severity, MDW by severity was compared and analyzed between the COVID-19 group and patients with acute respiratory infection in the non-COVID-19 group. MDW in each severity was significantly higher in the patients with COVID-19 than in the patients with acute respiratory infection in the non-COVID-19 group (Fig. 5).

### Relationship between MDW and other clinical parameters

Since the COVID-19 group was significantly younger than the patients with acute respiratory infection in the non-COVID-19 group, factors associated with MDW were evaluated. As a result, COVID-19 and CRP were found to be independent factors associated with MDW. Since age was not a factor associated with MDW, it was considered possible to compare MDW by severity between the COVID-19 group and patients with acute respiratory infection in the non-COVID-19 group (Table 3).

**Table 3:** Relationship between MDW and other clinical parameters: multiple regression analysis

Factors	95% confidence interval		*P* value
	Lower	Upper	
Age	− 0.0159	0.0211	NS
Gender	− 0.6675	0.8627	NS
COVID-19	3.3219	5.3098	< 0.001
Severity	− 0.3102	1.1857	NS
WBC	− 0.0001	0.0001	NS
CRP	0.1373	0.3081	< 0.001

WBC, white blood cell; CRP, C-reactive protein; MDW, monocyte distribution width

### Predictors of COVID-19

**Fig. 6:**
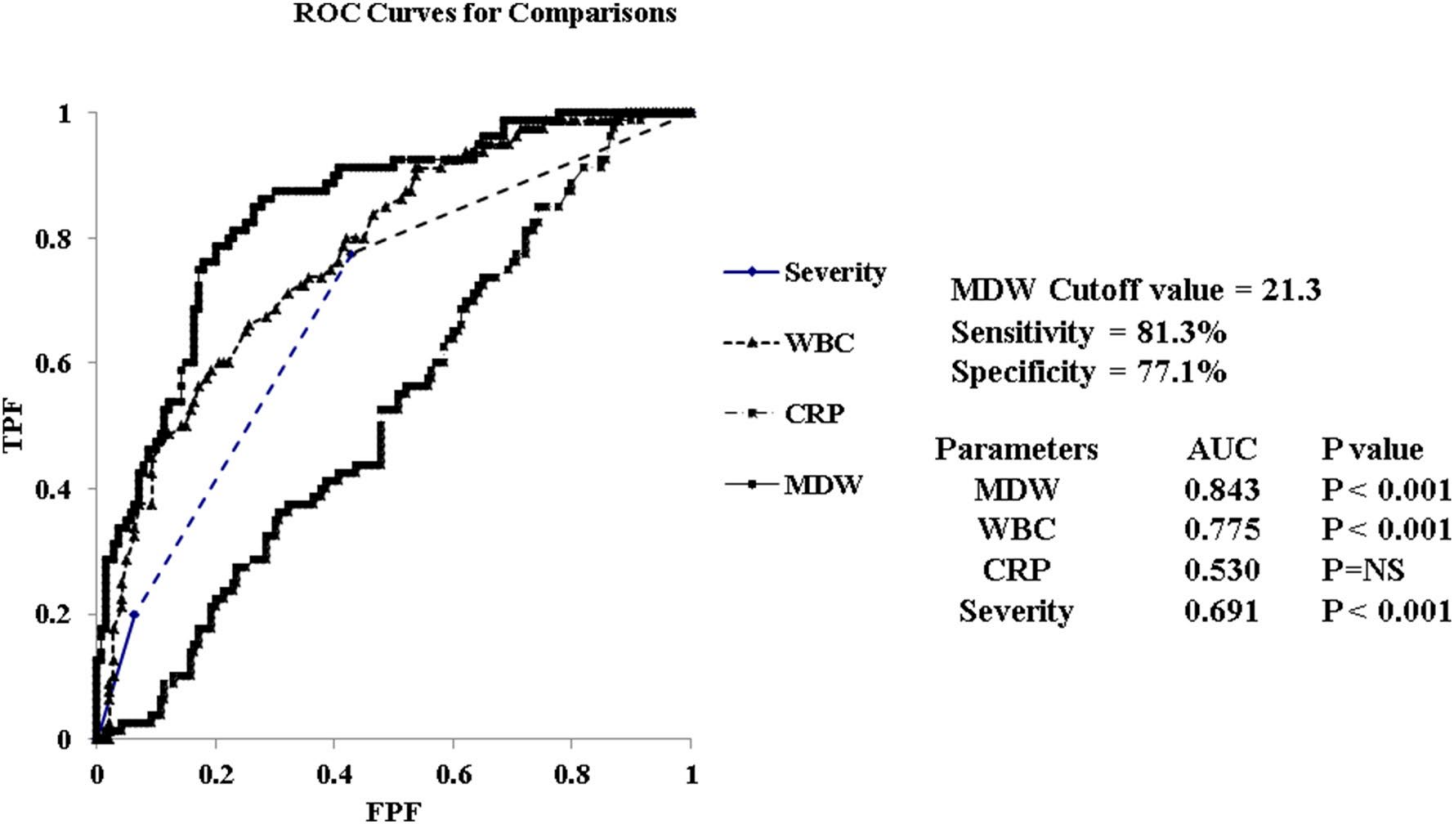
Results of the ROC analysis

Since the MDW levels were found to be strongly associated with COVID-19 patients, a multivariate analysis was performed to examine the predictors of COVID-19. Age remained an independent factor in the multivariate analysis because the COVID-19 patients in this population were clearly younger than the non-COVID-19 acute respiratory infection patients (Table 4). The ROC analysis suggested that MDW is a superior predictor of severity, WBC count and CRP level as a diagnostic predictor of COVID-19 (Fig. 6).

**Table 4:** Relationship between COVID-19 and other clinical parameters: multiple regression analysis

Factors	95% confidence interval		*P* value
	Lower	Upper	
Age	− 0.0718	− 0.0261	< 0.001
Gender	− 1.3614	0.5608	NS
Severity	1.2001	3.0856	< 0.001
WBC	− 0.0006	− 0.0001	< 0.001
NLR	− 0.1596	0.0681	NS
MDW	0.3289	0.7127	< 0.001
CRP	− 0.2939	− 0.0401	0.010

WBC, white blood cell; NLR, neutrophil-to-lymphocyte ratio; MDW, monocyte distribution width; CRP, C-reactive protein

## Discussion

In Japan, COVID-19 is diagnosed mainly by pathogenic genetic testing or antigen testing. Since genetic testing is more sensitive than antigen testing, pathogenic genetic testing is widely used for a definitive diagnosis of COVID-19 in Japan [24, 25]. However, dedicated equipment and skilled human resources are required to perform genetic testing, and strict infection control measures are required for the collection of nasopharyngeal swabs. There are other challenges. For example, it takes from several tens of minutes to several hours to obtain results [24], and the reagent cost per test is relatively high. Therefore, it is desirable that COVID-19 screening be performed easily in a routine clinical examination.

MDW generated by DxH 900, which was evaluated in this study, is a new cytometric parameter that reflects the changes in the monocyte cell volume caused by the activation of monocytes. The result is obtained in approximately one minute with routine CBC and WBC differential testing without the need for ordering additional tests. MDW values may be easily confirmed as a routine clinical examination. Therefore, MDW may be considered a promising screening test parameter for COVID-19 if its diagnostic performance for COVID-19 is superior.

Originally, monocytes played an important role in the innate immune system against infection and are believed to be involved in phagocytosis, antigen presentation, cytokine production, and activation of the acquired immune system. Additionally, activation of monocytes is considered to result in the expression of various functions and the diversity of morphology [9–14]. Similarly, neutrophil volume and distribution width are changed; however, Crouser et al. [1–3] evaluated MDWs in patients in the emergency department and reported that MDWs were superior in detecting sepsis patients and effective as the initial biomarker to aid in diagnosis.

In addition, Ognibene et al. [17] reported that MDWs may be useful as a diagnostic aid for COVID-19 based on an observational study analyzing 147 patients suspected to have COVID-19 presenting to the emergency department. The mean MDW in 41 SARS-CoV-2-positive patients was 27.3 ± 4.9 and that in 106 patients negative for SARS-CoV-2 was 20.3 ± 3.3 (*P* < 0.01). ROC analysis showed an AUC of 0.91, and MDW was quite effective in distinguishing SARS-CoV-2-positive patients from SARS-CoV-2-negative patients. It was reported that the sensitivity, specificity, positive predictive value, and negative predictive value were 98%, 65%, 51.9%, and 98.6%, respectively, at the MDW cut-off value of 20 [17]. Although our results were not superior to the results of this report, this study showed that MDW was highly effective in distinguishing SARS-CoV-2-positive patients from SARS-CoV-2-negative patients with an AUC of 0.844. At the MDW cut-off value of 21.3, the sensitivity and specificity were 81.3% and 78.2%, respectively. Although the specificity was inferior to that of antigen testing [26], it was considered that MDW might be used as a screening test in daily clinical practice.

In a multivariate analysis of the parameters affecting MDW, it was found that age was not related to MDW but that MDW levels were strongly associated with COVID-19. Lin et al. [18] examined inflammatory markers in COVID-19 patients and reported that MDW and NLR may be predictors of COVID-19. On the other hand, Badaki-Makun et al. [27] reported that the NLR was not a predictor of COVID-19. We performed a multivariate analysis of COVID-19 predictors, including the NLR. Severity, MDW, WBC and CRP may be predictors of COVID-19. Furthermore, the ROC analysis revealed that MDW is superior to severity, WBC count and CRP to predict COVID-19.

In the diagnosis of outpatients with fever, it is necessary to differentiate COVID-19 patients from non-COVID-19 patients with acute respiratory infection. We compared non-COVID-19 with acute respiratory infection by severity. The MDW in each severity was significantly higher in the COVID-19 group compared with the patients with acute respiratory infection in the non-COVID-19 group. These results suggest that COVID-19 is associated with more substantial morphological diversity of monocytes than non-COVID-19 patients with acute respiratory infection, suggesting that SARS-CoV-2 has a higher ability to activate cytokine production and adaptive immunity. Furthermore, COVID-19 and CRP were found to be independent factors associated with MDW, suggesting that MDW (cut-off value: 21.3) may be used as a screening test for COVID-19 in fever outpatients.

On the other hand, some patients with nonrespiratory infections also showed increased MDW values. This may be attributable to the fact that many severe patients were included in the nonrespiratory infection group because CRP was high. It is interesting that increased MDW values were observed in one patient with generalized herpes zoster, which is a viral infection. Since MDW is not a specific marker for COVID-19, it should be noted that MDW also increases in severe infections such as sepsis.

The limitation of this study was that the age of COVID-19 patients was younger than that of non-COVID-19 acute respiratory infections. Agnello et al. [28] examined MDW in 486 healthy blood donors and reported that MDW was not significantly associated with sex or age. Age did not remain a factor associated with MDW in our study. This study is a retrospective single-center study and does not include critically ill patients. In the future, prospective multicenter studies are necessary to address these issues.

## Conclusions

MDW can be performed easily in a short time as a routine clinical examination without using nasopharyngeal swab samples. Therefore, MDW is expected to be used as a screening test for COVID-19 before PCR testing. Furthermore, it is expected to be widely used clinically as a screening test at various medical institutions, such as small and medium-sized hospitals and clinics, where the introduction of genetic testing has been difficult.

## Data Availability

All data generated or analyzed during this study are included in this published article.

## Abbreviations

AUC: Area under the curve
CBC: Complete blood count
CE: Conformité Européenne
COVID-19: Coronavirus disease 2019
CRP: C-reactive protein
EDTA: Ethylenediaminetetraacetic acid
FDA: Food and Drug Administration
MDW: Monocyte distribution width
NLR: Neutrophil-to-lymphocyte ratio
NPV: Negative predictive value
PCR: Polymerase chain reaction
PCT: Procalcitonin
PPV: Positive predictive value
RBC: Red blood cell
ROC: Receiver operating characteristic
SARS-CoV-2: Severe acute respiratory syndrome coronavirus 2
SIRS: Systemic inflammatory response syndrome
SpO2: Oxygen saturation of peripheral artery
US: United States
WBC: White blood cell
WHO: World Health Organization
